# PROMOTING ACTIVE AGING THROUGH TAEKWONDO AMONG OLDER ADULTS

**DOI:** 10.1093/geroni/igae098.1174

**Published:** 2024-12-31

**Authors:** Minhwa Seo, Sua Im, Jinmoo Heo

**Affiliations:** Yonsei University, Seoul, Republic of Korea; Yonsei University, Seoul, Republic of Korea; Yonsei University, Seoul, Republic of Korea

## Abstract

With increasing longevity, the older adult’s population is growing, highlighting the importance of addressing issues related to active aging. In Korea, older adults actively participate in various leisure activities, including a specialized group practicing Taekwondo, a traditional Korean martial art, to promote active aging. This study aims to explore the role of Taekwondo engagement in active aging. Utilizing a phenomenological method design with semi-structured interviews, we explored how Taekwondo influences active aging. Participants aged 73 to 95, and each participant was diagnosed with at least one disease such as arthritis, stroke, or facial paralysis (n =12). Through in-depth interviews, three main themes and seven sub-themes emerged, all aligned with the WHO’s Active Aging model. The first theme highlights the promotion of diverse social participation through Taekwondo involvement, encompassing leisure, cultural engagement, community integration, and participation in competitions and performances. The second theme focuses on the positive impact on physical and mental health. Participants reported enhanced well-being, including reduced knee pain and improved cognitive function, following Taekwondo engagement and social interaction. The third theme emphasizes the security benefits associated with health promotion and participation in Taekwondo. Older adults were able to mitigate isolation through social connections and enhance their mobility and independence through physical activity. Overall, our findings underscore the potential of Taekwondo as a promising avenue for fostering active aging among older adults. This study contributes valuable insights for promoting health and well-being in aging populations.

